# Is it time for class I recommendation for sodium-glucose cotransporter-2 inhibitors in heart failure with mildly reduced or preserved ejection fraction?: An updated systematic review and meta-analysis

**DOI:** 10.3389/fcvm.2023.1046194

**Published:** 2023-02-07

**Authors:** Sukrit Treewaree, Narathorn Kulthamrongsri, Weerapat Owattanapanich, Rungroj Krittayaphong

**Affiliations:** ^1^Division of Cardiology, Department of Medicine, Faculty of Medicine Siriraj Hospital, Mahidol University, Bangkok, Thailand; ^2^Department of Pharmacology, Faculty of Medicine Siriraj Hospital, Mahidol University, Bangkok, Thailand; ^3^Division of Hematology, Department of Medicine, Faculty of Medicine Siriraj Hospital, Mahidol University, Bangkok, Thailand

**Keywords:** sodium-glucose cotransporter-2 inhibitors, heart failure with preserved ejection fraction, heart failure with mildly reduced ejection fraction, systematic review, meta-analysis

## Abstract

**Background:**

In heart failure with reduced ejection fraction (HFrEF), sodium-glucose cotransporter-2 (SGLT2) inhibitors were demonstrated to lower cardiovascular mortality (CV death) and hospitalization for heart failure (HHF); however, the advantages of SGLT2 inhibitors in heart failure with mildly reduced (HFmrEF) or preserved ejection fraction (HFpEF) are less clear. SGLT2 inhibitors were reported to enhance quality of life (QoL) in HFmrEF or HFpEF patients; however, the findings among studies are inconsistent.

**Objective:**

To conduct an updated systematic review and meta-analysis of recent data to assess the effect of SGLT2 inhibitors on cardiovascular outcomes and QoL in patients with HFmrEF or HFpEF.

**Method:**

Three databases were searched for studies that evaluated SGLT2 inhibitors and their effect on cardiovascular outcomes, including CV death, HHF, all-cause death, and the composite outcome of CV death, HHF, and urgent visit for heart failure (HF), and patient QoL (Kansas City Cardiomyopathy Questionnaire [KCCQ] score compared to baseline, and increase in KCCQ score ≥ 5 points) that were published during January 2000–August 2022. The meta-analysis was performed using the inverse variance method and random-effects model. INPLASY registration: INPLASY202290023.

**Results:**

Sixteen studies (9 recent RCTs) were included, and a total of 16,710 HFmrEF or HFpEF patients were enrolled. SGLT2 inhibitors significantly reduced composite cardiovascular outcome (CV death/HHF/urgent visit for HF; pooled hazard ratio [HR]: 0.80, 95% confidence interval [95%CI]: 0.74–0.86) and HHF alone (HR: 0.74, 95%CI: 0.67–0.82), but there was no significant reduction in CV death alone (HR: 0.93, 95%CI: 0.82–1.05). Benefit of SGLT2 inhibitors for decreasing CV death/HHF was observed across all subgroups, including left ventricular ejection fraction (LVEF) range, diabetes status, New York Heart Association functional class, and baseline renal function. For total HHF, SGLT2 inhibitors conferred benefit in both LVEF 50–60% (HR: 0.64, 95%CI: 0.54–0.76), and LVEF >60% (HR: 0.84, 95%CI: 0.71–0.98). Significant change was observed in the KCCQ-clinical summary score compared to baseline (mean difference: 1.33, 95%CI: 1.31–1.35), and meaningful improvement in QoL was shown across all 3 types of increase in KCCQ score ≥ 5 points.

**Conclusion:**

This study demonstrates the benefits of SGLT2 inhibitors for improving cardiovascular outcomes and QoL in HFmrEF or HFpEF patients.

## 1. Introduction

Heart failure (HF) is a clinical syndrome that comprises symptoms and signs of abnormal blood pumping and filling from or into the heart. HF is classified according to left ventricular ejection fraction (LVEF) into the 3 following groups: reduced ejection fraction (HFrEF; LVEF ≤40%), mildly reduced ejection fraction (HFmrEF; LVEF 41–49%), and preserved ejection fraction (HFpEF; LVEF ≥50%) (1, 2). The inclusion criteria of many previous clinical trials defined HFpEF as including patients with preserved ejection fraction or with mildly reduced ejection fraction. HF is a global health burden with over 60 million people reported to be affected by HF in 2017 (3). The prevalence of HF is increasing, and HFpEF is most commonly observed (4, 5). As the lifespan of people in most societies continues to increase, HF has emerged as a continuously growing global economic burden. The estimated global cost of treating HF in 2012 was 108 billion US dollars, and the reported direct cost per patient ranged from $800 to $30,000 per year (6–8).

There is robust evidence to support various treatments for reducing mortality and morbidity in patients with HFrEF; however, evidence specific to treatments for reducing mortality and morbidity in patients with HFmrEF or HFpEF is less clear (1, 2). Data from recent clinical trials suggest the benefit of sodium glucose co-transporter 2 (SGLT2) inhibitors as a potential treatment for patients with HFmrEF or HFpEF. The Empagliflozin Outcome Trial in Patients with Chronic Heart Failure with Preserved Ejection Fraction (EMPEROR-Preserved) trial reported a significantly reduced risk of composite cardiovascular death (CV death) or hospitalization for heart failure (HHF) in patients with HFmrEF or HFpEF compared to placebo; however, there was no significant effect on CV death alone or all-cause death alone (9).

Recent meta-analysis studies reported benefit of SGLT2 inhibitors for reducing HHF in HFmrEF or HFpEF, but the effects of SGLT2 inhibitors were inconsistent or none for reducing CV death alone or all-cause death alone (10–12). Furthermore, the benefits of SGLT2 inhibitors on HFpEF are not uniform throughout the LVEF spectrum and are mitigated in high LVEF (13).

Recent trials in SGLT2 inhibitors reported improved health status in patients with HFmrEF or HFpEF as measured by the Kansas City Cardiomyopathy Questionnaire (KCCQ) score; however, there are disparities in findings among studies (9, 14–17).

Given the recent publication of the data from the Dapagliflozin Evaluation to Improve the Lives of Patients with Preserved Ejection Fraction Heart Failure (DELIVER) trial (18), which was a large randomized double-blind trial that compared the effect of dapagliflozin versus placebo in patients with HFmrEF or HFpEF, an updated meta-analysis that focuses on the effect of SGLT2 inhibitor in patients with HFmrEF or HFpEF is urgently needed. Accordingly, the aim of this systematic review and meta-analysis was to evaluate data from recent studies that investigated the effect of SGLT2 inhibitors on patient quality of life (QoL) and cardiovascular (CV) outcomes, including CV death, hospitalization for HF (HHF), urgent visit for HF, and all-cause death, in patients with HFmrEF or HFpEF.

## 2. Methods

### 2.1. Data sources and searches

Systematic electronic searches of three online databases (OVID MEDLINE, Embase, and Cochrane CENTRAL) were independently conducted by two investigators (ST and NK) for articles published from 1 January 2000 to 28 August 2022. The search terms included keywords that maximized coverage of HF and SGLT2 inhibitors. Details specific to the search strategies used in this study are presented in Supplementary Data 1. The list of references in eligible studies, included studies, and studies of interest were manually screened to identify other suitable studies. This systematic review and meta-analysis was performed in accordance with the Preferred Reporting Items of Systematic Reviews and Meta-Analyses (PRISMA) 2020 guidelines (19) (Supplementary Data 2). The protocol for this study was approved by the Siriraj Institutional Review Board (SIRB) of the Faculty of Medicine Siriraj Hospital, Mahidol University, Bangkok, Thailand [COA no. 599/2565(IRB2)]. Written informed consent was not obtained from included patients due to the retrospective nature of this study.

### 2.2. Selection criteria and data extraction

The following criteria must have been satisfied for a study to be eligible for inclusion in the meta-analysis. First, the study must have been a randomized controlled trial (RCT) or a *post-hoc* analysis of an RCT that compared the outcomes of SGLT2 inhibitors with placebo or other hypoglycemic drugs for the treatment of HF. Second, the study must have reported at least one of the primary outcomes of interest, including CV death, HHF, all-cause death, or the composite outcome of CV death, HHF, and urgent visit for HF. Observational studies, case series, case reports, and reviews were excluded. The same two investigators that performed the database searches (ST, NK) independently determined the eligibility of identified studies. Any lack of agreement between those two investigators was resolved *via* the involvement of a third investigator (WO) until a consensus was reached. The data were independently extracted by the first two investigators (ST, NK) using a standardized data collection form, after which the accuracy and thoroughness of the data were verified by the third investigator (WO). The following data were collected: the name of the first author, year of publication, median follow-up time, intervention, baseline patient characteristics, and reported outcome(s) of interest.

### 2.3. Outcomes of interest

The primary outcome was the cardiovascular outcome, including CV death, HHF, all-cause death, and the composite outcome of CV death, HHF, and urgent visit for HF. The secondary outcomes were the change in the KCCQ score compared to baseline, an increase in the KCCQ score of ≥5 points, and total hospitalization due to HF (total HHF). The KCCQ has been used in many clinical trials to assess the health status of HF patients. The KCCQ comprises the following 7 domains: symptom frequency, symptom burden, symptom stability, physical limitations, social limitations, quality of life, and self-efficacy. The symptom frequency and symptom burden domain scores can be combined to generate the KCCQ-total symptom (KCCQ-TS) score. The KCCQ-TS score can be merged with the physical limitations domain score to generate the KCCQ-clinical summary (KCCQ-CS) score. The KCCQ-CS score can be combined with the social limitations domain score and quality of life domain score to generate the KCCQ-overall summary (KCCQ-OS) score. All scores are expressed on a 0-to-100 scale with a higher score indicating fewer symptoms, fewer limitations, and greater QoL (20).

### 2.4. Quality assessment of the included studies

The Cochrane Risk of Bias (RoB) 2 tool (The Cochrane Collaboration, London, United Kingdom) was used to evaluate the quality of included studies by two investigators (ST, NK), and any discrepancies were resolved *via* discussion and consensus between the two investigators (21).

### 2.5. Statistical analysis

The inverse variance method pooled the hazard ratios (HRs), odds ratios, mean differences, and 95% confidence intervals (95%CI) among the included studies (22). Cochran’s Q test was used to determine whether the proportion of ‘successes’ was equal across three or more groups. The statistical heterogeneity across the eligible studies was demonstrated using the prediction interval (23). A random effects model was used rather than a fixed effects model due to the high likelihood of between-study heterogeneity. A *value of p* of less than 0.05 was considered to reflect statistical significance. Review Manager 5.4 software from the Cochrane Collaboration was used for all statistical analyses. Publication bias was assessed using funnel plots. The study protocol was registered with the International Platform of Registered Systematic Review and Meta-analysis Protocols (INPLASY) network (registration number: INPLASY202290023).

## 3. Results

### 3.1. Study selection and risk of bias assessment

**Figure 1:**
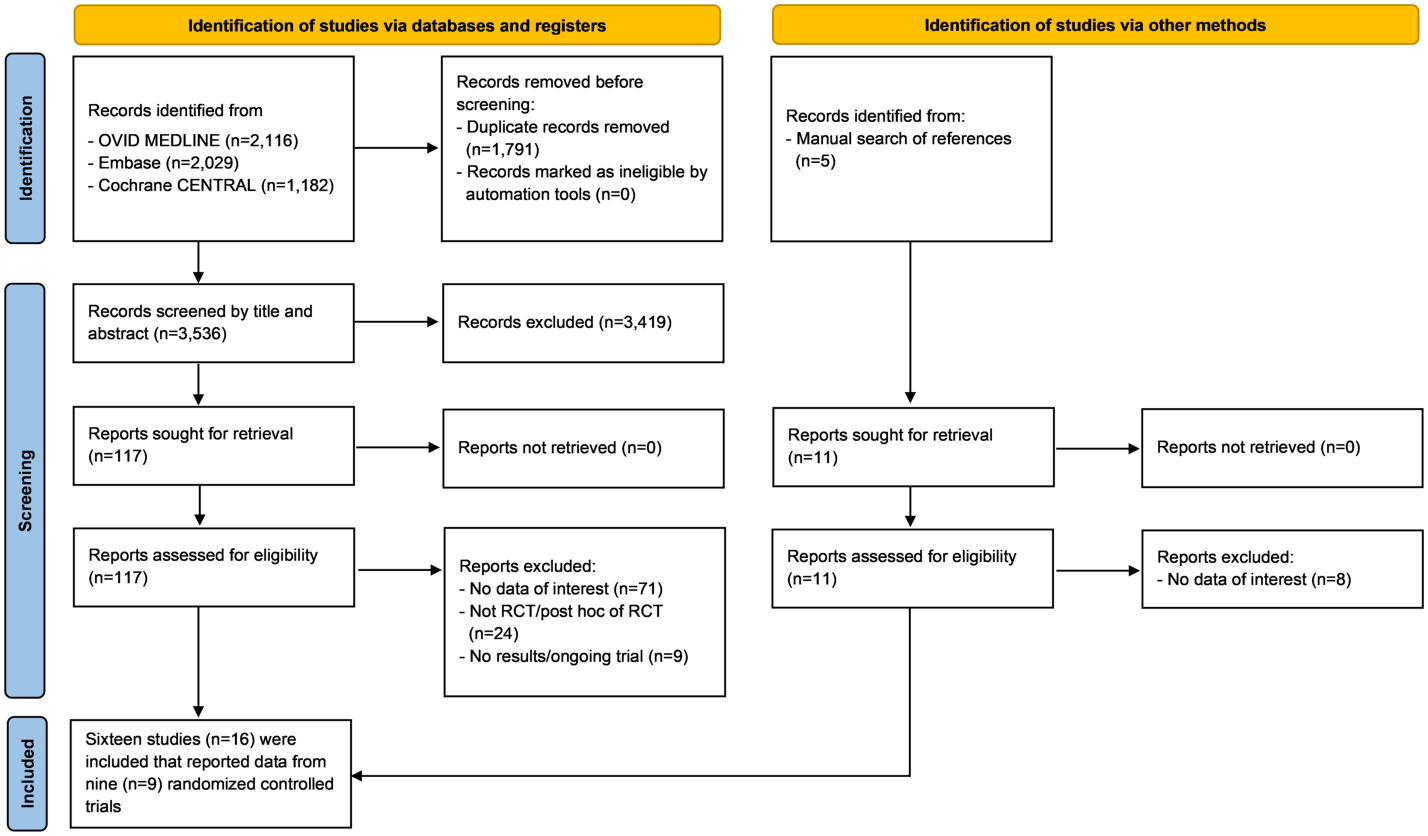
Preferred reporting items for systematic reviews and meta-analyses (PRISMA) flow diagram summarizing the systematic review and study selection protocol.

**Figure 2:**
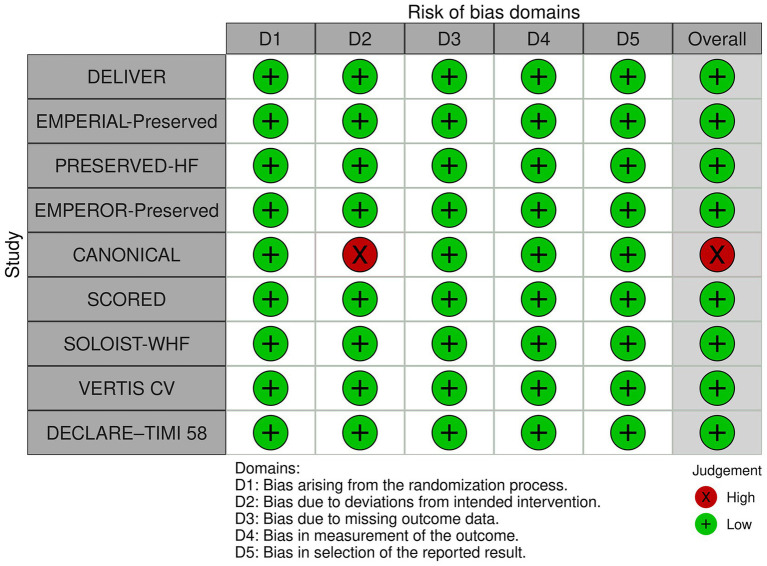
Quality assessment of the included randomized controlled trials using the Cochrane Risk of Bias 2 tool.

The initial search yielded 5,327 articles from the three online databases. After the removal of duplicates by the two authors who performed the searches (ST, NK), 3,547 records remained for screening by title and abstract, including 11 papers that were identified *via* a manual search of references. Those same two investigators independently reviewed the full text of 128 publications. Any disagreements between the two reviewing authors were resolved with the help and consultation of a third author (WO). One hundred and twelve articles were excluded for the following reasons: lacked data of interest (*n* = 79), not an RCT or a *post-hoc* analysis of an RCT (*n* = 24), and were ongoing trials or did not publish the results (*n* = 9). The remaining 16 articles that reported data from 9 recent RCTs were included in this meta-analysis (9, 14, 15, 24–36) (Figure 1) (19). The quality assessment using the RoB 2 tool (37) (The Cochrane Collaboration) is shown in Figure 2. We found no publication bias in study selection using funnel plots as shown in Supplementary Data 3.

Among the 112 excluded studies, five studies in HFpEF patients were also excluded from our meta-analysis. A *post-hoc* analysis of the Canagliflozin Cardiovascular Assessment Study (CANVAS) was excluded due to their having a different definition of the reported cardiac composite outcome (38). The Canagliflozin Impact on Health Status, Quality of Life, and Functional Status in Heart Failure (CHIEF-HF) trial, and the Empagliflozin in Patients Who Are in Hospital for Acute Heart Failure (EMPULSE) trial were excluded owing to the use of different statistics to report the change in KCCQ-TS (16, 17). The Prospective Comparison of Luseogliflozin and Alpha-glucosidase on the Management of Diabetic Patients with Chronic Heart Failure and Preserved Ejection Fraction (MUSCAT-HF) trial was excluded because it focused on echocardiographic outcome and had no outcome of interest (39). The Dapagliflozin Effect on Exercise Capacity Using a 6-min Walk Test in Patients with Heart Failure with Preserved Ejection Fraction (DETERMINE-preserved; NCT03877224) was excluded because its study results were publicly available only on the^1^ website, so it was an unpublished study.

### 3.2. Study and patient characteristics

A total of 16,710 HFmrEF or HFpEF patients from 9 RCTs were included in this study. The characteristics of the 9 randomized controlled trials that were included in this study, and from which data were analyzed and reported by the other 7 *post-hoc* studies included in this study are presented in Table 1. The median follow-up time ranged from 3 months in the Effect of Empagliflozin on Exercise Ability and Heart Failure Symptoms in Patients with Chronic Heart Failure (EMPERIAL)-Preserved trial and the Dapagliflozin in Preserved Ejection Fraction Heart Failure (PRESERVED-HF) trial to 50.4 months in the Multicenter Trial to Evaluate the Effect of Dapagliflozin on the Incidence of Cardiovascular Events (DECLARE–TIMI 58) (14, 15, 36). Except for the Canagliflozin Heart Failure with Preserved Ejection Fraction Study for Type 2 Diabetes Mellitus (CANONICAL) trial (34), which was an open-label randomized trial that compared canagliflozin to standard diabetic therapy, all studies were double-blind and placebo-controlled. Five trials (9, 14, 15, 33, 34) included participants with chronic HF, while the other four trials (24–26, 36) recruited type 2 diabetes patients with CV risk. The Effect of Sotagliflozin on Cardiovascular Events in Patients with Type 2 Diabetes Post Worsening Heart Failure (SOLOIST-WHF) trial (25) included type 2 diabetes patients who were hospitalized or had urgent heart failure visits. All trials excluded participants with renal impairment. Exclusion criteria for estimated glomerular filtration rate (eGFR) ranged from <20 ml/min/1.73 m^2^ to <30 ml/min/1.73 m^2^, or creatinine clearance (CrCl) <60 ml/min. Each trial used different LVEF cut points (range: 40–50%) for recruitment and/or for the analysis of data specific to HFpEF.

**Table 1:** The characteristics of the 9 randomized controlled trials that were included in this study, and from which data were analyzed and reported by the other 7 *post-hoc* studies included in this study.

Characteristics	DELIVER (N = 6,263)	EMPERIAL-Preserved (N = 315)	PRESERVED-HF (N = 324)	EMPEROR-Preserved (N = 5,988)	CANONICAL (N = 82)	SCORED (N = 10,584)	SOLOIST-WHF (N = 1,222)	VERTIS CV (N = 8,246)	DECLARE-TIMI 58 (N = 17,160)
Intervention	Dapagliflozin	Empagliflozin	Dapagliflozin	Empagliflozin	Canagliflozin	Sotagliflozin	Sotagliflozin	Ertugliflozin	Dapagliflozin
Year of publication	2022	2021	2021	2021	2021	2020	2020	2020	2018
Median follow-up time (months)	27.6	3.0	3.0	26.2	6.0	16.0	9.0	36.0	50.4
Key inclusion criteria	NYHA functional class II–IV; LVEF >40% and evidence of structural heart disease; Ambulatory or hospitalized patients	NYHA functional class II–IV; LVEF >40%; Evidence of structural heart disease or history of HF hospitalization within 12 months	NYHA functional class II–IV; LVEF ≥45%; Evidence of structural heart disease or history of acute treatment or hospitalization for HF within 12 months	NYHA functional class II–IV; LVEF >40%; Evidence of structural heart disease or history of HF hospitalization within 12 months	NYHA functional class II–III; LVEF ≥50% with history of HF; Type 2 diabetes with 6.5% ≤ HbA_1c_ < 10.0%	Type 2 diabetes with HbA_1c_ ≥ 7%; 25 ≤ eGFR ≤60 ml/min/1.73 m^2^; Having cardiovascular risk factor	Type 2 diabetes; hospitalized or visit due to worsening HF; Chronic treatment with loop diuretic for >30 days; Previous diagnosis of HF (>3 months); Randomized when hemodynamically stable within 3 days of discharge	Type 2 diabetes with 7.0% ≤ HbA_1c_ ≤ 10.5%; Evidence or a history of atherosclerosis	Type 2 diabetes; Established cardiovascular disease and/or multiple cardiovascular risk factors
Key exclusion criteria	eGFR <25 ml/min/1.73 m^2^	eGFR <20 ml/min/1.73 m^2^	eGFR <20 ml/min/1.73 m^2^	eGFR <20 ml/min/1.73 m^2^	Severe renal dysfunction or hemodialysis; NYHA functional class IV	History of dialysis within 1 year; End-stage HF	eGFR <30 ml/min/1.73 m^2^; End-stage HF	eGFR <30 ml/min/1.73 m^2^; NYHA functional class IV	CrCl <60 ml/min; NYHA functional class IV
Definition of preserved EF	>40%	>40%	≥45%	>40%	≥50%	≥50%	≥50%	>45%	≥45%
Number of patients with HFpEF	6,263 (100%)	315 (100%)	324 (100%)	5,988 (100%)	82 (100%)	1,667 (15.8%)	256 (20.9%)	1,007 (12.2%)	808 (4.7%)
Reported outcomes of interest*	1, 2, 3, 4, 5, 6	6	3, 6	1, 2, 3, 5, 6	2, 3	5	5	1, 2, 3, 5	1, 2, 3, 5

* Reported outcomes of interest: 1 – CV death; 2 – HHF; 3 – all-cause death; 4 – worsening HF; 5 – cardiac composite; 6 – KCCQ score. Abbreviations: CANONICAL, CANagliflOziN heart faIlure with preserved ejection fraCtion study for type 2 diAbetes meLlitus trial; CrCl, creatinine clearance; CV, cardiovascular; DECLARE-TIMI 58, Dapagliflozin Effect on CardiovascuLAR Events trial; DELIVER, Dapagliflozin Evaluation to improve the LIVEs of patients with pReserved ejection fraction heart failure trial; EF, ejection fraction; eGFR, estimated glomerular filtration rate; EMPERIAL-Preserved, effect of EMPagliflozin on ExeRcise ability and heart failure symptoms In patients with chronic heArt faiLure trial; EMPEROR-Preserved, EMPagliflozin outcomE tRial in Patients With chrOnic heaRt Failure With Preserved Ejection Fraction trial; HbA_1c_, glycated hemoglobin; HF, heart failure; HFpEF, heart failure with preserved ejection fraction; HHF, hospitalization due to heart failure; KCCQ score, Kansas City Cardiomyopathy Questionnaire score; LVEF, left ventricular ejection fraction; NYHA, New York Heart Association; PRESERVED-HF, dapagliflozin in PRESERVED ejection fraction Heart Failure trial; SCORED, effect of Sotagliflozin on CardiOvascular and Renal Events in patients with type 2 Diabetes and moderate renal impairment who are at cardiovascular risk trial; SOLOIST-WHF, effect of SOtagLiflOzin on cardiovascular events In patientS with Type 2 diabetes post Worsening Heart Failure trial; VERTIS CV, eValuation of ERTugliflozin effIcacy and Safety CardioVascular outcomes trial.

Data specific to the outcomes of interest were extracted from the DECLARE-TIMI 58, SOLOIST-WHF, Cardiovascular Outcomes Following Ertugliflozin Treatment in Type 2 Diabetes Mellitus Participants with Vascular Disease (VERTIS-CV), EMPERIAL-preserved, Empagliflozin Outcome Trial in Patients with Chronic Heart Failure with Preserved Ejection Fraction (EMPEROR-Preserved), PRESERVED-HF, CANONICAL, Effect of Sotagliflozin on Cardiovascular and Renal Events in Patients with Type 2 Diabetes and Moderate Renal Impairment Who Are at Cardiovascular Risk (SCORED), and DELIVER trials (9, 14, 15, 24–26, 33, 34, 36).

In the present study, in addition to including data from studies that recruited HFpEF patients only (EMPEROR-Preserved, PRESERVED-HF, and DELIVER), we also included data from studies that recruited both HFpEF and non-HFpEF patients, including the DECLARE-TIMI 58 trial (36), which included diabetes mellitus (DM) patients with and without history of heart failure, and the EMPERIAL trial (14), which recruited HF patients with HFrEF or HFpEF.

The majority of patients in all included trials were male, except for the PRESERVED-HF trial (15). The mean body mass index (BMI) of patients in most trials classified them as overweight or class 1 obesity (40). In all studies, a higher proportion of patients were in New York Heart Association (NYHA) functional class I-II than in functional class III-IV. Approximately half of the patients included in the present study were patients with DM that were enrolled in trials that had HF as part of the inclusion criteria. Patient baseline characteristics from the 9 randomized controlled trials included in this study, and from which data were analyzed and reported by the other 7 *post-hoc* studies included in this study are presented in Table 2.

**Table 2:** Patient baseline characteristics from the 9 randomized controlled trials included in this study, and from which data were analyzed and reported by the other 7 *post-hoc* studies included in this study.

Characteristics	DELIVER (N = 6,263)		EMPERIAL-Preserved (N = 315)		PRESERVED-HF (N = 324)		EMPEROR-Preserved (N = 5,988)		CANONICAL (N = 82)		SCORED (N = 10,584)		SOLOIST-WHF (N = 1,222)		VERTIS CV (N = 8,246)		DECLARE-TIMI 58 (N = 17,160)	
Comparison	Drug	Placebo	Drug	Placebo	Drug	Placebo	Drug	Placebo	Drug	Standard diabetic therapy	Drug	Placebo	Drug	Placebo	Drug	Placebo	Drug	Placebo
Number of patients	3,131	3,132	157	158	162	162	2,997	2,991	42	40	5,292	5,292	608	614	680 ^a^	327 ^a^	8,582	8,578
Mean ± SD or median (IQR) age (years)	71.8 ± 9.6	71.5 ± 9.5	74 (68–79)	75 (68–81)	69 (64–77)	71 (63–78)	71.8 ± 9.3	71.9 ± 9.6	76.5 ± 6.4	75.9 ± 5.8	69 (63–74)	69 (63–74)	69 (63–76)	70 (64–76)	63.8 ± 8.3	64.7 ± 8.2	63.9 ± 6.8	64.0 ± 6.8
Female, n (%)	1,364 (43.6%)	1,383 (44.2%)	70 (44.6%)	66 (41.8%)	92 (56.8%)	92 (56.8%)	1,338 (44.7%)	1,338 (44.6%)	NA (33.3%)	NA (32.5%)	2,347 (44.3%)	2,407 (45.5%)	198 (32.6%)	214 (34.9%)	NA (34.4%)	NA (36.7%)	3,171 (36.9%)	3,251 (37.9%)
Mean ± SD or median (IQR) BMI (kg/m^2^)	29.8 ± 6.2	29.9 ± 6.1	30.1 (26.5–34.2)	28.8 (26.1–32.8)	35.1 (30.4–41.8)	34.6 (29.7–40.4)	29.77 ± 5.8	29.90 ± 5.9	24.7 ± 3.6	25.2 ± 3.7	31.9 (28.1–36.2)	31.7 (28.0–36.1)	30.4 (26.3–34.3)	31.1 (27.3–34.5)	32.6 ± 5.3	32.9 ± 5.3	32.1 ± 6.0	32.0 ± 6.1
NYHA functional class, *n* (%)																		
I-II	2,314 (73.9%)	2,399 (76.6%)	117 (74.5%)	126 (79.7%)	96 (59.3%)	90 (55.6%)	2,435 (81.2%)	2,452 (82.0%)	88.10%	95.0%					89.6%	93.3%		
III-IV	817 (26.1%)	732 (23.4%)	39 (24.8%)	32 (20.3%)	65 (40.1%)	72 (44.4%)	562 (18.8%)	539 (18.0%)	11.90%	5.0%					7.2%	4.6%		
Mean ± SD or median (IQR) LVEF (%)	54.0 ± 8.6	54.3 ± 8.9	53 (45–58)	53 (46–59)	60 (55–65)	60 (54–65)	54.3 ± 8.8	54.3 ± 8.8	61.1 ± 7.8	61.9 ± 7.6	60 (51–64)	60 (51–65)	35 (28–47)	35 (28–45)				
Median (IQR) NT-ProBNP, pg./ml			966 (572–1,653)	843 (407–1,913)	641 (373–1,210)	710 (329–1,449)	994 (501–1,740)	946 (498–1,725)			196 (75–565)	198 (75–561)	1,817 (855–3,659)	1,741 (843–3,582)				
Diabetes mellitus, n (%)	1,401 (44.7%)	1,405 (44.9%)	86 (54.8%)	75 (47.5%)	90 (55.6%)	91 (56.2%)	1,466 (48.9%)	1,472 (49.2%)	100%	100%	100%	100%	100%	100%	100%	100%	100%	100%
Mean ± SD or median (IQR) eGFR (mL/min/1.73 m^2^)	61 ± 19	61 ± 19	54.5 (41–70)	58.5 (44–71.5)	56 (42–69)	54 (41–69)	60.6 ± 19.8	60.6 ± 19.9	57.8 ± 14.2	56.0 ± 13.8	44.4 (37–51.3)	44.7 (37–51.5)	49.2 (39.5–61.2)	50.5 (40.5–64.6)			85.4 ± 15.8	85.1 ± 16.0

a Data from HFmrEF or HFpEF population. Abbreviations: BMI, body mass index; CANONICAL, CANagliflOziN heart faIlure with preserved ejection fraCtion study for type 2 diAbetes meLlitus trial; DECLARE-TIMI 58, Dapagliflozin Effect on CardiovascuLAR Events trial; DELIVER, Dapagliflozin Evaluation to improve the LIVEs of patients with pReserved ejection fraction heart failure trial; eGFR, estimated glomerular filtration rate; EMPERIAL-Preserved, effect of EMPagliflozin on ExeRcise ability and heart failure symptoms In patients with chronic heArt faiLure trial; EMPEROR-Preserved, EMPagliflozin outcomE tRial in Patients With chrOnic heaRt Failure With Preserved Ejection Fraction trial; IQR, interquartile range; LVEF, left ventricular ejection fraction; NA, not available; NT-ProBNP, N-terminal pro B-type natriuretic peptide; NYHA, New York Heart Association; PRESERVED-HF, dapagliflozin in PRESERVED ejection fraction Heart Failure trial; SCORED, effect of Sotagliflozin on CardiOvascular and Renal Events in patients with type 2 Diabetes and moderate renal impairment who are at cardiovascular risk trial; SD, standard deviation; SOLOIST-WHF, effect of SOtagLiflOzin on cardiovascular events In patientS with Type 2 diabetes post Worsening Heart Failure trial; VERTIS CV, eValuation of ERTugliflozin effIcacy and Safety CardioVascular outcomes trial.

### 3.3. SGLT2 Inhibitors reduce the incidence of CV outcomes

**Figure 3:**
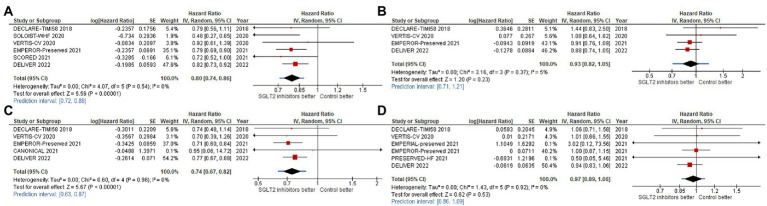
Forest plots of studies that investigated **(A)** CV death or HHF or urgent visit for heart failure (HF); **(B)** cardiovascular **(CV)** death; **(C)** hospitalization for heart failure (HHF); and, **(D)** all-cause death compared between patients receiving sodium-glucose cotransporter-2 (SGLT2) inhibitors and controls among all heart failure with mildly reduced (HFmrEF) or preserved ejection fraction (HFpEF) patients.

For our primary outcome, SGLT2 inhibitors reduced composite CV outcome comprising CV death or HHF or urgent visit for HF (hazard ratio [HR]: 0.80, 95% confidence interval [95%CI]: 0.74–0.86, prediction interval: 0.72–0.89; Figure 3A). The same trends were observed for CV death alone with significant heterogeneity (HR: 0.93, 95%CI: 0.82–1.05, prediction interval: 0.71–1.21; Figure 3B), for HHF alone (HR: 0.74, 95%CI: 0.67–0.82, prediction interval: 0.63–0.87; Figure 3C), and for all-cause death alone (HR: 0.97, 95%CI: 0.89–1.06, prediction interval: 0.86–1.09).

**Figure 4:**
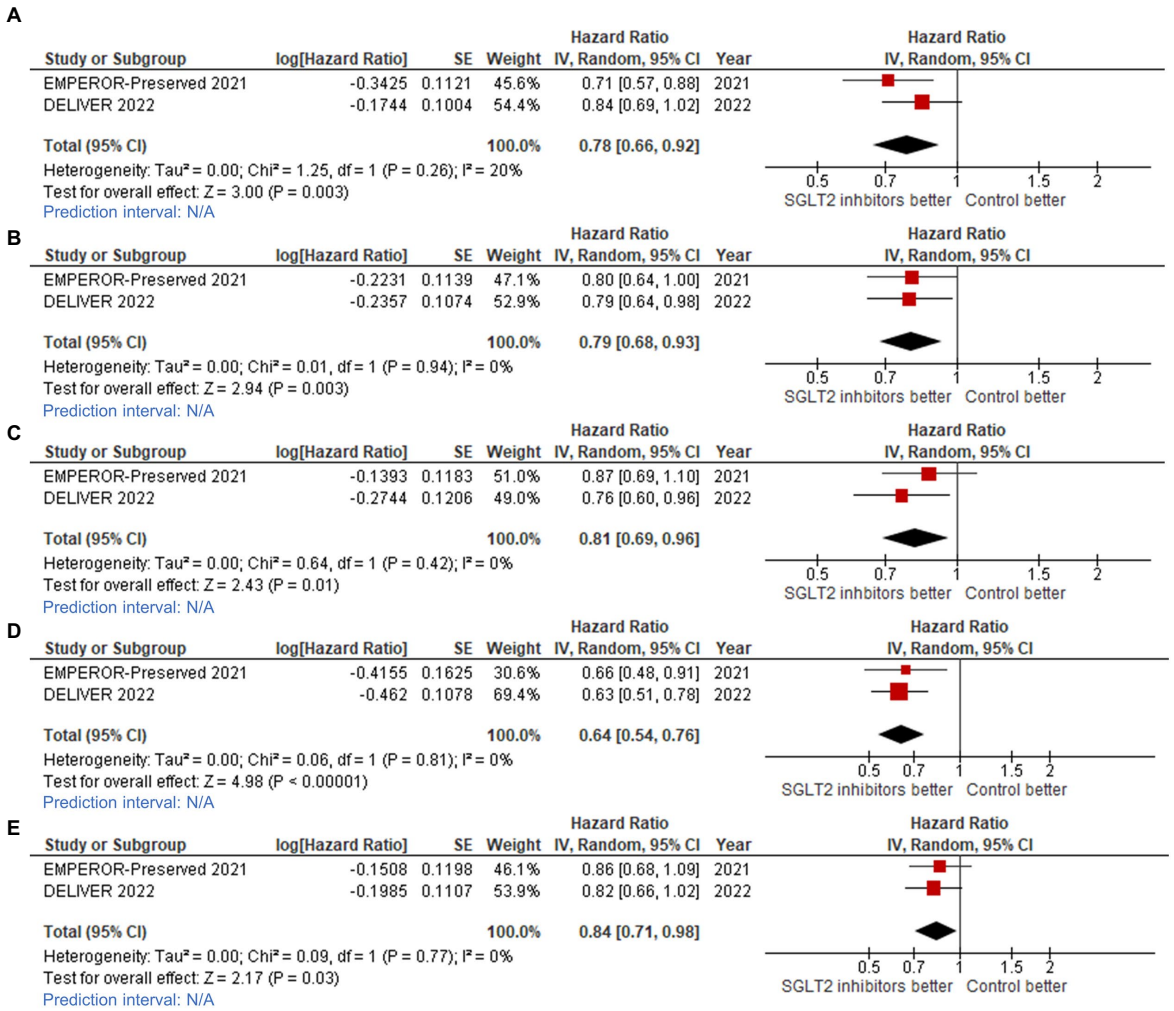
Forest plots of studies that compared patients receiving sodium-glucose cotransporter-2 (SGLT2) inhibitors versus controls among patients with **(A)** ejection fraction (EF) 40–50% in cardiovascular (CV) death/hospitalization for heart failure (HHF) outcome; **(B)** EF 50–60% in CV death/HHF outcome; **(C)** EF >60% in CV death/HHF outcome; **(D)** EF 50–60% in total HHF outcome; and, **(E)** EF >60% in total HHF outcome.

**Figure 5:**
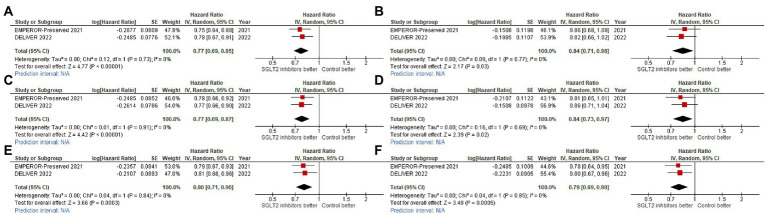
Forest plots of studies that compared cardiovascular (CV) death/hospitalization for heart failure (HHF) outcome among patients receiving sodium-glucose cotransporter-2 (SGLT2) inhibitors versus controls in patients with **(A)** New York Heart Association functional class II (NYHA II); **(B)** NYHA III-IV; **(C)** estimated glomerular filtration rate (eGFR) <60 ml/min/1.73 m2; **(D)** eGFR ≥60 ml/min/1.73 m2; **(E)** diabetes mellitus (DM); and, **(F)** non-DM.

The effect of SGLT2 inhibitors on the composite CV outcome, including CV death or first HHF or urgent visit for HF, was also found to be consistent across 12 clinically relevant subgroups (Figures 4, 5).

Statistically significant benefit of SGLT2 inhibitors for reducing CV death/HHF was observed across ejection fraction groups, as follows: LVEF 40–50% (HR: 0.78, 95%CI: 0.66–0.92, prediction interval: N/A; Figure 4A); LVEF 51–60% (HR: 0.79, 95%CI: 0.68–0.93, prediction interval: N/A; Figure 4B); and, LVEF >60% (HR: 0.81, 95%CI: 0.69–0.96, prediction interval: N/A; Figure 4C).

We found consistent benefit across NYHA functional classification groups, as follows: NYHA functional classification I or II (HR: 0.77, 95%CI: 0.67–0.85, prediction interval: N/A; Figure 5A), and NYHA functional classification III or IV (HR: 0.84, 95%CI: 0.71–0.98, prediction interval: N/A; Figure 5B). We also found consistent benefit across baseline renal function groups [eGFR <60% (HR: 0.77, 95%CI: 0.69–0.87, prediction interval: N/A; Figure 5C), and ≥ 60% (HR: 0.84, 95%CI: 0.73–0.97, prediction interval: N/A; Figure 5D)], and across DM status groups [DM (HR: 0.80, 95%CI: 0.71–0.90, prediction interval: N/A; Figure 5E), and non-DM (HR: 0.79, 95%CI: 0.69–0.90, prediction interval: N/A; Figure 5F)].

We also observed that SGLT2 inhibitors reduced the total number of HHF across ejection fraction groups, as follows: LVEF 50–60% (HR: 0.64, 95%CI: 0.54–0.76, prediction interval: N/A; Figure 4D), and LVEF >60% (HR: 0.84, 95%CI: 0.71–0.98, prediction interval: N/A; Figure 4E).

### 3.4. SGLT2 inhibitors improve health status and QoL

**Figure 6:**
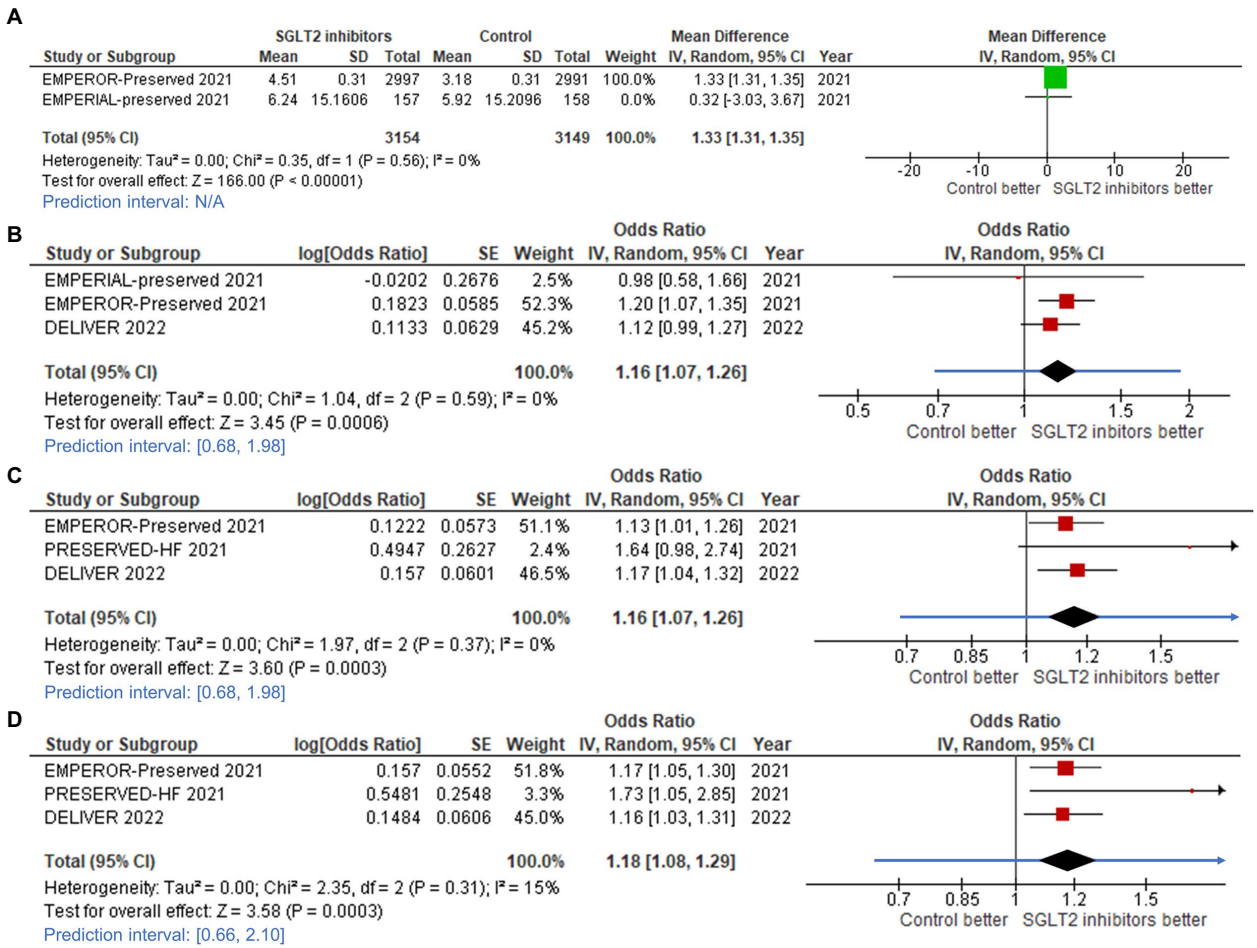
Forest plots of studies that evaluated **(A)** change in Kansas City Cardiomyopathy Questionnaire (KCCQ) – clinical summary score; **(B)** increased KCCQ-total symptom (TS) score; **(C)** increased KCCQ-clinical summary (CS) score; and, **(D)** increased KCCQ-overall summary (OS) score compared between patients receiving sodium-glucose cotransporter-2 (SGLT2) inhibitors and controls among all heart failure with mildly reduced (HFmrEF) or preserved ejection fraction (HFpEF) patients.

More participants in the SGLT2 inhibitor groups experienced clinically significant improvements as measured by the 3 types of KCCQ scores (TS, CS, and OS) when compared to controls, as demonstrated by the mean change in KCCQ-CS score compared to baseline (mean difference: 1.33, 95%CI: 1.31–1.35, prediction interval: N/A; Figure 6A), KCCQ-TS score increase of ≥5 points from baseline (odds ratio [OR]: 1.16, 95%CI: 1.07–1.26, prediction interval: 0.68–1.98; Figure 6B), KCCQ-CS score increase of ≥5 points from baseline (OR: 1.16, 95%CI: 1.07–1.26, prediction interval: 0.68–1.98; Figure 6C), and KCCQ-OS score increase of ≥5 points from baseline (OR: 1.18, 95%CI: 1.08–1.29, prediction interval: 0.66–2.10; Figure 6D).

## 4. Discussion

The results of this meta-analysis demonstrate that SGLT2 inhibitors, including dapagliflozin, empagliflozin, canagliflozin, and sotagliflozin, significantly or compellingly reduced CV outcome, including any type of death, HHF, or urgent visit due to HF with no or minimal evidence of heterogeneity among trials. The various subanalyses that we performed also revealed that SGLT2 inhibitors improve CV outcomes across LVEF range groups, DM status groups, baseline renal function groups, and NYHA functional class groups.

This meta-analysis also demonstrated that SGLT2 inhibitors improve the health status of patients with HFpEF as measured and supported by both the change in the mean KCCQ-CS score compared to baseline, and the increase in the KCCQ score ≥ 5 points. The 5-point threshold was considered to reflect a clinically meaningful improvement in health status in many studies, and was also reported to be associated with improvement in functional capacity (41, 42). The benefit of SGLT2 inhibitors on health status has been demonstrated in HFrEF patients but remains controversial in HFpEF. (43) The mean change in the KCCQ-CS score in the PRESERVED-HF and EMPEROR-Preserved studies indicated statistically significant benefits of SGLT2 inhibitors for improving health status, but no statistically significant benefit was found in the EMPERIAL-Preserved trial (9, 14, 15). Incorporating data from the recent DELIVER trial (33, 35), the present meta-analysis found statistically and clinically meaningful improvement in all 3 KCCQ combination scores (KCCQ-TS, KCCQ-CS, and KCCQ-OS). These results strongly suggest that HFmrEF or HFpEF patients that are prescribed SGLT2 inhibitors experience improved health status and QoL.

The mechanisms behind the benefits of SGLT2 inhibitors in HFpEF patients are under investigation. Diastolic dysfunction, subtle systolic dysfunction, atrial dysfunction, and endothelial dysfunction are the main contributors to HFpEF (44, 45). There have been studies that demonstrate that SGLT2 inhibitors alleviate diastolic dysfunction in both HFrEF and HFpEF animal models, which may explain the CV benefits and improved QoL in HFpEF patients (46, 47).

HFpEF patients were recognized to have more non-cardiac comorbidities than HFrEF patients, which play vital roles in the management and prognosis of HFpEF patients. In addition, atrial fibrillation was common in HFpEF patients and associated with increased adverse CV events (48–50). It is probable that SGLT2 inhibitors could have varying effects on HFpEF patients with different comorbidities. However, there was no statistically significant difference in the effects of SGLT2 inhibitors on reducing the composite of CV mortality or HHF between HFpEF patients with and without the following comorbidities: age, diabetes, obesity (BMI ≥ 30 kg/ m^2^), impaired renal function (eGFR <60 ml/min/1.73 m^2^), and history of atrial fibrillation (35). The lack of differences in the effect of SGLT2 inhibitors might be owing to no difference between groups or the inadequate statistical power in subgroup analysis.

To our knowledge, this is the first meta-analysis to study the effect of SGLT2 inhibitors in HFmrEF or HFpEF patients that analyzed extractable data from all of the previously conducted RCTs on this study topic, including DECLARE-TIMI 58, VERTIS CV, SOLOIST-WHF, SCORED, CANONICAL, PRESERVED-HF, EMPERIAL-Preserved, and the 2 most recent large trials – EMPEROR-Preserved and DELIVER. Some earlier meta-analyses that studied the outcome of SGLT2 inhibitors in heart failure patients did not focus solely on HFmrEF or HFpEF patients (12, 51). Moreover, the meta-analyses that did set forth to focus on HFmrEF or HFpEF population did not extensively analyze the same outcome or specific subgroups as our meta-analysis had done (10, 52). Our study also included an increase of at least 5 points in KCCQ score, which reflects new clinical impact on the aspect of patient QoL.

Despite the clinical benefit of SGLT2 inhibitors in HFmrEF or HFpEF relative to CV death and all-cause death being demonstrated in this study, the improvement in those two parameters was not statistically significantly increased. Similarly, previous RCTs reported the benefit of SGLT2 inhibitors for reducing CV outcome and improving patient QoL even though their data did not show statistically significant difference between study and controls. Accordingly, the overriding aim of the present meta-analysis was to compile the current data from focused RCTs, and to use that amplified statistical power to evaluate the effect of SGLT-2 inhibitors on CV outcomes and patient QoL among patients with HFmrEF or HFpEF. The current weaker class IIa recommendations for SGLT-2 inhibitors among HFmrEF and HFpEF patients, were based on the previously reported non-statistically significant improvements in CV outcomes among HFmrEF or HFpEF patients; however, the guideline recommendations for SGLT-2 inhibitor use in HFrEF patients are class I recommendations (1, 2). The present meta-analysis also sheds important light on questions about the efficacy of SGLT2 inhibitors in each specific subgroup of HFmrEF or HFpEF patients. This meta-analysis together with recent data from the DELIVER trial (33) demonstrates the clear and undeniable positive impact of SGLT2 inhibitors on essential clinical events and symptom burden in patients with HFmrEF or HFpEF across various subgroups. Because SGLT2 inhibitors have a favorable but not statistically significant benefit in reducing CV death and all-cause death as individual outcome, further investigation is needed to elevate the recommendation for SGLT2 inhibitors in HFmrEF or HFpEF from class IIa to class I. These findings suggest that SGLT2 inhibitors should be considered for treating patients with HFmrEF or HFpEF. We hope that ongoing studies that are focusing on various outcomes of SGLT2 inhibitors, such as NCT04249778, the DAPPER study (JPRN-jRCTs051180135), EUCTR 2020–004832-48-GB, and EUCTR2015-005715-32-SE, will yield greater insights that will further improve the management of patients with HFmrEF or HFpEF, and support future guideline updates.

Since none of the studies included in this meta-analysis compared one SGLT2 inhibitor against another SGLT2 inhibitor. An RCT study, which was not included in our meta-analysis due to lack of an outcome of interest, showed that Sotagliflozin has greater effects on some of the metabolic and antidiabetic effects when compare with Empagliflozin, which might have implications for the clinical outcome (53). Thus, we cannot deny the possibility of differences in clinical efficacy and safety between and among the different SGLT2 inhibitors.

Despite this meta-analysis demonstrating significant benefits of SGLT2 inhibitors in HFmrEF or HFpEF by pooling data from 9 clinical trials, there are some limitations that must be disclosed and discussed. First, there were variations in the duration of time between the baseline KCCQ and the final KCCQ that ranged from 12 weeks in the PRESERVED-HF and EMPERIAL-Preserved trials to 52 weeks in the EMPEROR-Preserved trial (9, 14, 15). The results of all 3 of these trials favored the use of SGLT2 inhibitors over placebo, except the difference between study and control was statistically significant in the PRESERVED-HF and EMPEROR-Preserved trials, but non-significant in the EMPERIAL-Preserved trial. This difference among groups may be due to the difference in follow-up duration. Second, individual participant-level data from each study were not available to us, so we resorted to using publicly accessible data. As such, some outcomes or subgroup factors might be more accurately represented in pooled analysis if participant-level data were available. The PRESERVED-HF, EMPERIAL-Preserved, CHIEF-HF, and EMPULSE trials (14–16, 54) reported changes in KCCQ-OS score and KCCQ-TS score from baseline using different statistics, including mean difference, Hodges-Lehmann median difference, and least square mean difference. Which means that these data could not be directly included in the pooled analysis. Moreover, two studies (55, 56) reported their methods for estimating the mean or effect size, but they were limited by their data distribution assumptions. We, therefore, decided to omit the aforementioned outcome data to avoid misinterpretation. Among the 9 RCTs that generated all of the data used in all 16 included studies, only the DELIVER, EMPEROR-Preserved, and DECLARE-TIMI 58 trials had LVEF subgroup range data available, and there were differences in the reported cut point used among those 3 studies. DECLARE-TIMI 58 stratified LVEF into 45–54% and ≥ 55% (30), whereas DELIVER stratified LVEF into 41–49%, 50–59%, and ≥ 60% (9, 35). Third, due to discrepancies in exclusion criteria between trials, our study may not identify some subtypes of HFpEF. The DELIVER, EMPEROR-Preserved, SOLOIST-WHF, EMPERIAL-preserved, and PRESERVED-HF trials excluded infiltrative and hypertrophic obstructive cardiomyopathy, while other trials in our analysis did not mention this. Therefore, some cases with cardiac amyloidosis and hypertrophic cardiomyopathy (HCM) might be included in this analysis. Because treatment and prognosis differ between HFpEF caused by amyloidosis or HCM and other etiologies, more investigation into each subtype of HFpEF is warranted (1, 57, 58). Finally, renal endpoint was excluded from our meta-analysis. Only the EMPEROR-Preserved trial reported composite renal outcomes, which consisted of chronic dialysis, renal transplantation, sustained decrease in eGFR of ≥40% or sustained eGFR <15 ml/min/1.73 m^2^ in patients with a baseline eGFR ≥30 ml/min/1.73 m^2^, or < 10 ml/min/1.73 m^2^ in patients with a baseline eGFR <30 ml/min/1.73 m^2^ (9, 32). The MUSCAT-HF trial reported only percentage change in eGFR (39). Most of the other trials reported adverse renal events only with no clear definition. This finding may influence future trials to integrate renal outcomes into their study design. No adjustment for multiplicity of testing was made for subgroup analyses.

## 5. Conclusion

The results of this systematic review and meta-analysis demonstrated the benefit of SGLT2 inhibitors for significantly reducing the risk of the composite of CV death, HHF, or urgent visit for HF compared to placebo in patients with HFmrEF or HFpEF, but their benefit for reducing CV death alone and all-cause death alone could not be established. We also found that SGLT2 inhibitors improve KCCQ scores, which translates to improved patient QoL. The results of this meta-analysis indicate the universal beneficial impact of SGLT2 inhibitors in patients with HFmrEF or HFpEF irrespective of baseline ejection fraction, renal status, NYHA functional class, and diabetes status. Taken together, these results suggest that SGLT2 inhibitors should be considered for treating patients with HFmrEF or HFpEF to improve patient outcomes and QoL. Further study into the effects of SGLT2 inhibitors on CV death, all-cause mortality, and different background therapies, as well as on less-studied outcomes, such as renal outcome, respiratory outcome, and neurological outcome are warranted, Study of the effect of SGLT2 inhibitors in various metabolic and hemodynamic scenarios is also recommended.

## Data availability statement

The data analyzed in this study is subject to the following licenses/restrictions: All datasets generated for this study are available from the corresponding author upon reasonable request. Requests to access these datasets should be directed to rungroj.kri@mahidol.ac.th.

## Author contributions

All authors participated in the design of the study. ST and NK performed the search and study selection processes. WO analyzed the data and generated the forest plots. ST, NK, and WO wrote the manuscript. ST created all other figures and tables. WO and RK critically assessed the manuscript for important intellectual content. All authors contributed to the article and approved the submitted version.

## Conflict of interest

The authors declare that the research was conducted in the absence of any commercial or financial relationships that could be construed as a potential conflict of interest.

## Publisher’s note

All claims expressed in this article are solely those of the authors and do not necessarily represent those of their affiliated organizations, or those of the publisher, the editors and the reviewers. Any product that may be evaluated in this article, or claim that may be made by its manufacturer, is not guaranteed or endorsed by the publisher.

## Footnotes

^1^ clinicaltrials.gov
