# Supplementary material for: Is it time for class I recommendation for sodium-glucose cotransporter-2 inhibitors in heart failure with mildly reduced or preserved ejection fraction?: An updated systematic review and meta-analysis
Source: Front Cardiovasc Med. 2023 Feb 7;10:1046194. doi: 10.3389/fcvm.2023.1046194 (PMC9941559; doi:10.3389/fcvm.2023.1046194)
Supplement: Supplementary file 1 [file Data_Sheet_1.DOCX]

**Supplementary Data 1.** Search strategy for the systematic review

**Ovid MEDLINE(R)**

1 exp Heart Failure/ or heart failure*.mp.

2 ((heart or cardi* or myocard* or ventric* or systolic or diastolic or LV or RV) and (failure* or insufficien* or dysfunction* or decompensat*)).mp. [mp=title, book title, abstract, original title, name of substance word, subject heading word, floating sub-heading word, keyword heading word, organism supplementary concept word, protocol supplementary concept word, rare disease supplementary concept word, unique identifier, synonyms]

3 (HF or CHF or HF*EF or LVD or RVD or LVF or RVF).mp. [mp=title, book title, abstract, original title, name of substance word, subject heading word, floating sub-heading word, keyword heading word, organism supplementary concept word, protocol supplementary concept word, rare disease supplementary concept word, unique identifier, synonyms]

4 1 or 2 or 3

5 Sodium-Glucose Transporter 2 Inhibitor.mp. or exp Sodium-Glucose Transporter 2 Inhibitors/

6 ((Sodium-Glucose or Sodium Glucose) and (transporter 2 or cotransporter 2 or transporter-2 or cotransporter-2 or co-transporter 2 or co-transporter-2) and (Inhibitor* or antago*)).mp. [mp=title, book title, abstract, original title, name of substance word, subject heading word, floating sub-heading word, keyword heading word, organism supplementary concept word, protocol supplementary concept word, rare disease supplementary concept word, unique identifier, synonyms]

7 ((sglt2 or sglt 2 or sglt-2) and (Inhibitor* or antago* or inh)).mp. [mp=title, book title, abstract, original title, name of substance word, subject heading word, floating sub-heading word, keyword heading word, organism supplementary concept word, protocol supplementary concept word, rare disease supplementary concept word, unique identifier, synonyms]

8 (sglt2i or sglt2 i).mp.

9 *gliflozin/ or *gliflozins/

10 canagliflozin.mp. or exp Canagliflozin/

11 dapagliflozin.mp.

12 empagliflozin.mp.

13 ertugliflozin.mp.

14 ipragliflozin.mp.

15 luseogliflozin.mp.

16 tofogliflozin.mp.

17 sergliflozin.mp.

18 sotagliflozin.mp.

19 remogliflozin.mp.

20 5 or 6 or 7 or 8 or 9 or 10 or 11 or 12 or 13 or 14 or 15 or 16 or 17 or 18 or 19

21 4 and 20

22 randomized controlled trial.pt.

23 controlled clinical trial.pt.

24 randomized.ab.

25 placebo.ab.

26 drug therapy.fs.

27 randomly.ab.

28 trial.ab.

29 groups.ab.

30 22 or 23 or 24 or 25 or 26 or 27 or 28 or 29

31 exp animals/ not humans.sh.

32 30 not 31

33 21 and 32

**Cochrane CENTRAL**

#1 MeSH descriptor: [Heart Failure] explode all trees

#2 (((heart or cardi* or myocard* or ventric* or systolic or diastolic or LV or RV) and (failure* or insufficien* or dysfunction* or decompensat*))) (Word variations have been searched)

#3 ((HF or CHF or HF*EF or LVD or RVD or LVF or RVF)) (Word variations have been searched)

#4 #1 or #2 or #3 (Word variations have been searched)

#5 MeSH descriptor: [Sodium-Glucose Transporter 2 Inhibitors] explode all trees

#6 ((("Sodium-Glucose" or "Sodium Glucose") and ("transporter 2" or "cotransporter 2" or "transporter-2" or "cotransporter-2" or "co-transporter 2" or "co-transporter-2") and (Inhibitor* or antago*))) (Word variations have been searched)

#7 (((sglt2 or "sglt 2" or "sglt-2") and (Inhibitor* or antago* or inh))) (Word variations have been searched)

#8 ((sglt2i or "sglt2 i")) (Word variations have been searched)

#9 (*gliflozin or *gliflozins) (Word variations have been searched)

#10 (canagliflozin or dapagliflozin or empagliflozin or ertugliflozin or ipragliflozin or luseogliflozin or tofogliflozin or sotagliﬂozin or remogliﬂozin or sergliﬂozin) (Word variations have been searched)

#11 #5 or #6 or #7 or #8 or #9 or #10 (Word variations have been searched)

#12 #4 and #11

#13 #12 in Trials

**Embase**

#01 'heart failure'/exp OR 'heart failure'

#02 ('heart'/exp OR heart OR cardi* OR myocard* OR ventric* OR systolic OR diastolic OR lv OR rv) AND (failure* OR insufficien* OR dysfunction* OR decompensat*)

#03 'hf'/exp OR hf OR chf OR hf*ef OR 'lvd'/exp OR lvd OR rvd OR lvf OR rvf

#04 #1 OR #2 OR #3

#05 'sodium glucose cotransporter 2 inhibitor'/exp OR 'sodium glucose cotransporter 2 inhibitor'

#06 ('sodium-glucose' OR 'sodium glucose') AND ('transporter 2' OR 'cotransporter 2' OR 'transporter-2' OR 'cotransporter-2' OR 'co-transporter 2' OR 'co-transporter-2') AND (inhibitor* OR antago*)

#07 ('sglt2' OR 'sglt 2' OR 'sglt-2') AND (inhibitor* OR antago* OR 'inh'/exp OR inh)

#08 sglt2i OR 'sglt2 i'

#09 'gliflozin'/exp OR gliflozin OR 'gliflozins'/exp OR gliflozins

#10 'canagliflozin'/exp OR canagliflozin OR 'dapagliflozin'/exp OR dapagliflozin OR 'empagliflozin'/exp OR empagliflozin OR 'ertugliflozin'/exp OR ertugliflozin OR 'ipragliflozin'/exp OR ipragliflozin OR 'luseogliflozin'/exp OR luseogliflozin OR 'tofogliflozin'/exp OR tofogliflozin OR sotagliﬂozin OR remogliﬂozin OR sergliﬂozin

#11 #5 OR #6 OR #7 OR #8 OR #9 OR #10

#12 #4 AND #11

#13 #4 AND #11 AND [english]/lim

#14 'randomized controlled trial'/de

#15 'controlled clinical trial'/de

#16 random*:ti,ab,tt

#17 'randomization'/de

#18 'intermethod comparison'/de

#19 placebo:ti,ab,tt

#20 compare:ti,tt OR compared:ti,tt OR comparison:ti,tt

#21 (evaluated:ab OR evaluate:ab OR evaluating:ab OR assessed:ab OR assess:ab) AND (compare:ab OR compared:ab OR comparing:ab OR comparison:ab)

#22 (open NEXT/1 label):ti,ab,tt

#23 ((double OR single OR doubly OR singly) NEXT/1 (blind OR blinded OR blindly)):ti,ab,tt

#24 'double blind procedure'/de

#25 (parallel NEXT/1 group*):ti,ab,tt

#26 crossover:ti,ab,tt OR 'cross over':ti,ab,tt

#27 ((assign* OR match OR matched OR allocation) NEAR/6 (alternate OR group OR groups OR intervention OR interventions OR patient OR patients OR subject OR subjects OR participant OR participants)):ti,ab,tt

#28 assigned:ti,ab,tt OR allocated:ti,ab,tt

#29 (controlled NEAR/8 (study OR design OR trial)):ti,ab,tt

#30 volunteer:ti,ab,tt OR volunteers:ti,ab,tt

#31 'human experiment'/de

#32 trial:ti,tt

#33 #14 OR #15 OR #16 OR #17 OR #18 OR #19 OR #20 OR #21 OR #22 OR #23 OR #24 OR #25 OR #26 OR #27 OR #28 OR #29 OR #30 OR #31 OR #32

#34 ((random* NEXT/1 sampl* NEAR/8 ('cross section*' OR questionnaire* OR survey OR surveys OR database OR databases)):ti,ab,tt) NOT ('comparative study'/de OR 'controlled study'/de OR 'randomised controlled':ti,ab,tt OR 'randomized controlled':ti,ab,tt OR 'randomly assigned':ti,ab,tt)

#35 'cross‐sectional study' NOT ('randomized controlled trial'/de OR 'controlled clinical trial'/de OR 'controlled study'/de OR 'randomised controlled':ti,ab,tt OR 'randomized controlled':ti,ab,tt OR 'control group':ti,ab,tt OR 'control groups':ti,ab,tt)

#36 'case control*':ti,ab,tt AND random*:ti,ab,tt NOT ('randomised controlled':ti,ab,tt OR 'randomized controlled':ti,ab,tt)

#37 'systematic review':ti,tt NOT (trial:ti,tt OR study:ti,tt)

#38 nonrandom*:ti,ab,tt NOT random*:ti,ab,tt

#39 'random field*':ti,ab,tt

#40 ('random cluster' NEAR/4 sampl*):ti,ab,tt

#41 review:ab AND review:it NOT trial:ti,tt

#42 'we searched':ab AND (review:ti,tt OR review:it)

#43 'update review':ab

#44 (databases NEAR/5 searched):ab

#45 (rat:ti,tt OR rats:ti,tt OR mouse:ti,tt OR mice:ti,tt OR swine:ti,tt OR porcine:ti,tt OR murine:ti,tt OR sheep:ti,tt OR lambs:ti,tt OR pigs:ti,tt OR piglets:ti,tt OR rabbit:ti,tt OR rabbits:ti,tt OR cat:ti,tt OR cats:ti,tt OR dog:ti,tt OR dogs:ti,tt OR cattle:ti,tt OR bovine:ti,tt OR monkey:ti,tt OR monkeys:ti,tt OR trout:ti,tt OR marmoset*:ti,tt) AND 'animal experiment'/de

#46 'animal experiment'/de NOT ('human experiment'/de OR 'human'/de)

#47 #34 OR #35 OR #36 OR #37 OR #38 OR #39 OR #40 OR #41 OR #42 OR #43 OR #44 OR #45 OR #46

#48 #33 NOT #47

#49 #12 AND #48
