# Supplementary material for: Is it time for class I recommendation for sodium-glucose cotransporter-2 inhibitors in heart failure with mildly reduced or preserved ejection fraction?: An updated systematic review and meta-analysis
Source: Front Cardiovasc Med. 2023 Feb 7;10:1046194. doi: 10.3389/fcvm.2023.1046194 (PMC9941559; doi:10.3389/fcvm.2023.1046194)

**Supplementary Data 3.** Funnel plots showing distribution of studies for following outcomes: (A) CV death or HHF or urgent visit for HF; (B) CV death; (C) HHF; and (D) all-cause death


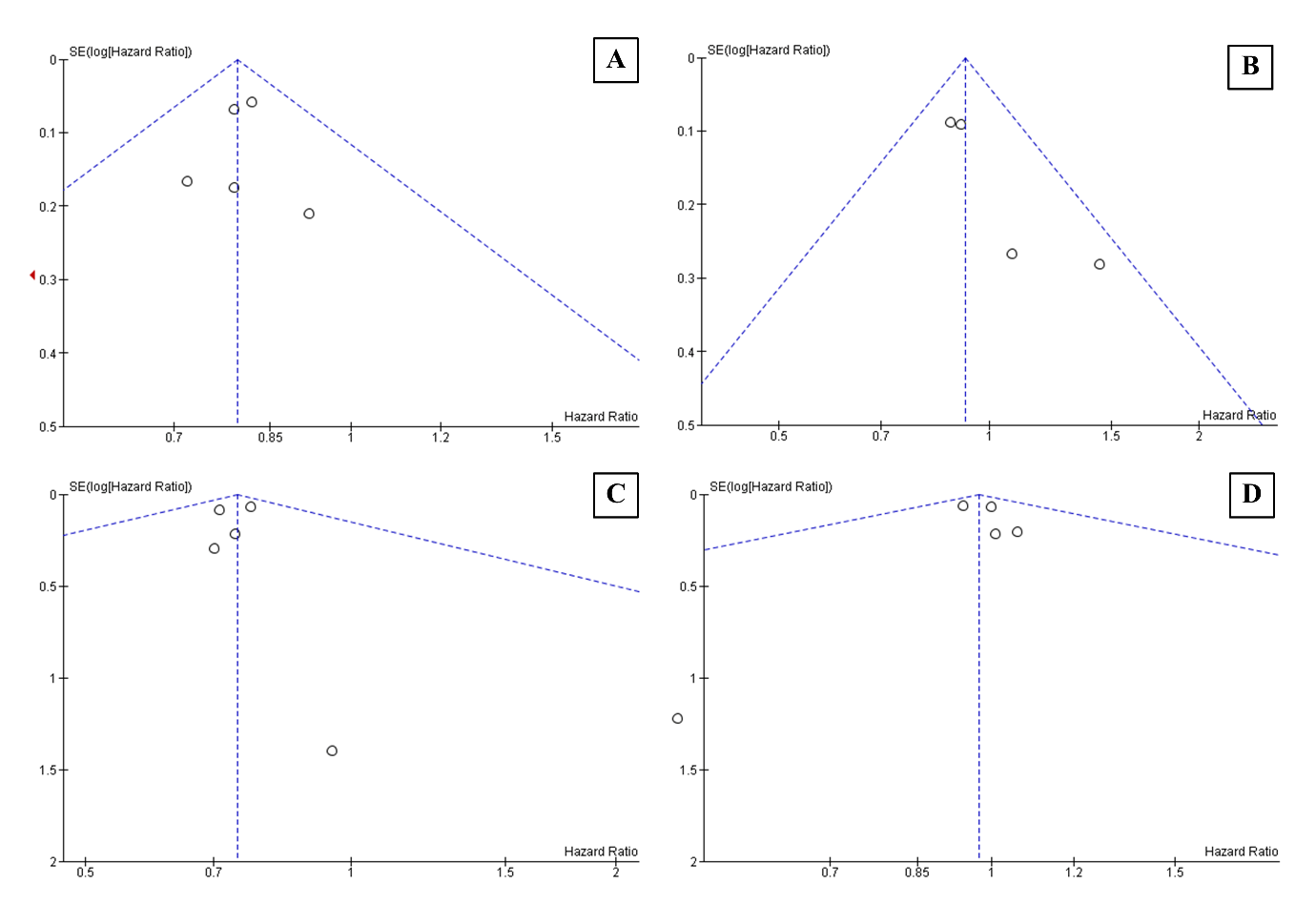

Supplement: Supplementary file 2 [file Table_1.DOCX]
